# Rescue of a decapentaplegic hypomorphic wing growth phenotype by expression of the signaling ligands Hedgehog, Semaphorin a1, and Wnt homolog 2 in *Drosophila melanogaster*

**DOI:** 10.1093/g3journal/jkag202

**Published:** 2026-07-27

**Authors:** Anthony Percival-Smith, Cooper Brabrook

**Affiliations:** Department of Biology, The University of Western Ontario, 1151 Richmond St., London, Ontario, Canada N6A 5B7; Department of Biology, The University of Western Ontario, 1151 Richmond St., London, Ontario, Canada N6A 5B7

**Keywords:** phenotypic nonspecificity, transcription factor redundancy, Hedgehog, Semaphorin, wing growth, Wnt ortholog 2, signaling ligand, Dros2026

## Abstract

An expectation of a hypothesis that proposes cell-to-cell signaling pathways are redundant due to the redundancy of pathway terminal transcription factors (TFs) was tested by screening 35 signaling ligands for rescue of a decapentaplegic (dpp) hypomorphic wing growth phenotype. Three examples of partial rescue were identified: Hedgehog (HH), Semaphorin 1a (SEMA1A), and Wnt ortholog 2 (WNT2). HH overexpression with *dppGAL4* may increase the expression of DPP from the hypomorphic *dpp* alleles. However, SEMA1A and WNT2 did not phenocopy the ectopic expression of HH or DPP, and neither SEMA1A nor WNT2 were required for wing growth, suggesting substitution of DPP for restoration of wing growth. The WNT2 rescue was dependent on the Frizzled 4 WNT receptor excluding the possibility that WNT2 weakly binds the DPP receptor. Although examples of phenotypic nonspecificity of signaling ligand function were identified, this is an expectation, and not direct proof, of the hypothesis of TF redundancy.

## Introduction

Cell-to-cell signaling is essential for the development and physiology of multicellular and single-celled organisms. One general sequence of information flow common to many signaling pathways is the specific interaction of a ligand with a transmembrane receptor on the surface of the cell followed by the activation of an intracellular pathway that leads to changes in the activity of pathway terminal transcription factors (TFs), thereby altering gene expression in the cell. The protein encoded by the *Drosophila dpp* gene is a cell-to-cell signaling ligand of the transforming growth factor β ligand family and the *Drosophila* ortholog of vertebrate bone morphogenetic proteins (Padgett et al. 1987). DPP is required for many different developmental processes of which a limited list includes embryonic dorsal ventral axis formation, axis formation of the developing adult leg, wing growth and patterning, eye formation, and gonad development (Posakony et al. 1990; Chanut and Heberlein 1997; Xie and Spradling 1998). In both embryonic dorsal ventral axis formation and wing morphogenesis, DPP is proposed to function as a concentration-dependent morphogen (Ferguson and Anderson 1992; Nellen et al. 1996). However, subsequent studies of the role of DPP spreading during wing morphogenesis do not support DPP acting as a simple concentration-dependent morphogen in wing pattern formation (Akiyama and Gibson 2015; Harmansa et al. 2015; Matsuda and Affolter 2023). The protein complex consisting of the DPP ligand bound to the Thickvein Punt transmembrane co-receptors phosphorylates the TF Mothers against decapentaplegic (dpp) (MAD), and the phosphorylated MAD forms a protein complex with another TF Medea that together enter the nucleus to alter gene expression (Padgett et al. 1998).

The functional redundancy of TFs results in the phenomenon of phenotypic nonspecificity, which is the substitution of 1 TF^a^ with that of another TF^b^ for the rescue of the TF^a^ phenotype (Percival-Smith 2017; Percival-Smith et al. 2023). Combining the observations that signal transduction pathways affect TF activity and that TF activity is redundant suggests the possibility that cell-to-cell signaling pathways are also phenotypically nonspecific. Phenotypic nonspecificity of cell-to-cell signaling would be the substitution of 1 signaling ligand **a** by another unrelated signaling ligand **b** for rescue of the signaling ligand **a** phenotype. This rescue is the hypothetical result of the TFs affected by the presence/absence of the signaling ligands **a** or **b** being functionally redundant. This expectation of the TF redundancy hypothesis was assessed by screening a set of *Drosophila* signaling factors expressed with a *dppGAL4* driver for wing growth rescue of a *dpp* hemizygous, hypomorphic mutant combination (Spencer et al. 1982).

## Materials and methods

### *Drosophila* husbandry and the screen

Flies were maintained at 24 °C and 60% humidity. The flies were reared in 20-mL vials containing corn meal media [1% {w/v} *Drosophila* grade agar, 6% {w/v} sucrose, 10% {w/v} cornmeal, 1.5% {w/v} yeast, and 0.375% {w/v} 2-methyl hydroxybenzoate]. The genotypes of the stocks used in this study are given in Supplementary Table 1. The stocks were used in standard *Drosophila* crosses to assemble the specific genotypes used in this study. FlyBase was first screened for genes that express secreted signaling ligands using the gene ontology (GO) term “receptor ligand activity,” and this subset of genes was then screened for available stocks at the Bloomington and FlyORF *Drosophila* stock centers with GAL4 upstream activation sequence (UAS) insertions expressing the various signaling ligands on the third chromosome (Bischof et al. 2013; Öztürk-Çolak et al. 2024). The UAS fly stocks were used to create the common genotype *y w; dpp^d5^/CyO; P or M{UASX, w^+^}*, where X is the ligand expressed. For the screen, virgins of this common genotype were crossed with *y w; dpp^d6^/L; P{dppGAL4, w^+^}/+* males, which were generated by crossing virgin *y w; CyO/L; P{dppGAL4, w^+^}/TM6B* females with *dpp^d6^/CyO* males. The *y w; dpp^d5^/dpp^d6^; P or M{UASX, w^+^}/P{dppGAL4, w^+^* progeny were screened for rescue of the dpp hypomorphic wing phenotype (*P{dppGAL4, w^+^}* progeny have dark red eyes).

### Imaging rescue and quantification of wing area

For imaging the rescued wings with scanning electron microscopy, flies were first critical point dried and then sputter coated. The images were collected using a scanning electron microscope. For wing area measurements, adult wings were plucked and placed in 70% ethanol. The wings were mounted on slides in Hoyer's mountant (Wieschaus and Nusslein-Volhard 1986). The wing area was collected on a Zeiss AxioImager Z1 microscope using Zen imaging software (Carl Zeiss Canada Inc.). DPP expression was knocked down in the progeny of *w; P{nubGAL4}, P{UASmCherry}; P{UASdeGradFP*} X *y^1^ v^1^; P{TRiP.JF01371}attP2* (Supplementary Table 1).

### Analysis of the requirement of the Frizzled 4 receptor

To assess whether the Frizzled 4 (FZ4) receptor was required for rescue of the dpp phenotype by expression of Wnt ortholog 2 (WNT2), the *fz4^3–1^* null mutant allele (McElwain et al. 2011) was used to generate *y w fz4^3–1^*; *dpp^d5^/CyO; M{UASWnt2, w^+^}* virgin females that were crossed with *y w; dpp^d6^/CyO; P{dppGAL4, w^+^}/+* males. The *y w fz4^3–1^; dpp^d5^/dpp^d6^; M{UASWnt2, w^+^}/; P{dppGAL4, w^+^}* male progeny were assessed for rescue.

### RNA interference

Male *w; P{nubGAL4}, P{UASmCherry}; P{UASdeGradFP*} flies were crossed with *w^1118^, P{UASDcr2}; Df(3L)Ly, sens^Ly-1^/TM3, Sb^1^* virgin females, and male *P{UASDcr-2}, w^1118^; P{nubGAL4}/TM3 Sb* progeny collected and crossed with *y^1^ v^1^; P{TRiP.JF03231}attP2*, *y^1^ sc v^1^ sev^21^; P{TRiP.HM05221}attP2*, *y^1^ sc v^1^ sev^21^; P{TRiP.HMS01307}attP2* and *y^1^ sc v^1^ sev^21^; P{TRiP.GL01870}attP40* virgin females (Supplementary Table 1; Ni et al. 2009). For measuring the wing area, the wings were plucked from female flies that had neither third nor second chromosome balancers. For quantification of *PLEXA* mRNA, *w; P{nubGAL4}, P{UASmCherry}; P{UASdeGradFP*} males were crossed with either *y^1^ sc v^1^ sev^21^; P{TRiP.HM05221}attP2* or *y^1^ sc v^1^ sev^21^; P{TRiP.GL01870}attP40* and wing imaginal discs dissected from late third instar larval progeny.

### Quantitative PCR

Male and female larvae were distinguished by the characteristic sexually dimorphic size of their gonads. A total of 10 to 20 wing discs were dissected from late third instar larvae and total RNA extracted with a RNeasy mini kit (QIAGEN). The RNA was reversed transcribed with SuperScript IV (Thermo Fisher). The sequences of the primers used for qPCR are given in Supplementary Table 2. The qPCR was performed with the GB-Amp PowerGreen qPCR mix (GeneBio Systems) and run in a CFX Connect real-time PCR (Bio-Rad).

### Generation of virtual in situ images

The integration of wing disc single-cell RNA-seq data with a set of in situ hybridization gene expression patterns allows the generation of spatial gene expression patterns (Everetts et al. 2021). The data and code provided in Everetts et al. (2021) were used to generate virtual in situ images of gene expression.

### Statistical analysis

The data were assessed for normality and equal variance with QQ plots and plotting the residuals, respectively, using the Prism program for statistical analyses (GraphPad). If the data (untransformed or transformed) met the criteria for an ordinary ANOVA, it was performed, and multiple pairwise comparison post hoc analyses were performed with Tukey's or Dunnett's test. If the criteria for an ordinary ANOVA were not met, a nonparametric Kruskal–Wallis test (ANOVA of ranks) was performed, followed by multiple pairwise comparisons using Dunn's post hoc test.

## Results and discussion

### The screen for rescue of a hypomorphic dpp wingless phenotype

FlyBase was searched with the GO term “receptor ligand activity” in 2022 identifying 106 genes (Öztürk-Çolak et al. 2024). These genes were screened for the availability of a GAL4 *UAS* construct inserted on the third chromosome in a *Drosophila* stock center. Forty-five *UAS* stocks were identified for 39 genes (Supplementary Table 1). Forty of 45 crosses representing 35 genes yielded progeny of the correct genotype to assess rescue (Table 1). Relative to wild-type wings, the *dpp^d5^/dpp^d6^* wing phenotype is a strongly reduced wing that still retains the wing costal cell and alula (Fig. 1a, b, and f; Jürgens et al. 2024). Expression of DPP from a *UAS* construct using an imaginal disc-specific *dppGAL4* driver rescues wing growth (Fig. 1f). When expressed in a *dpp^d5^/dpp^d6^* background (Fig. 1b) using an imaginal disc-specific *dppGAL4* driver, 3 *UAS* constructs expressing the coding regions derived from 3 genes, *hedgehog* (*hh)*, *Semaphorin-a1* (*Sema-a1)*, and *Wnt homolog 2* (*Wnt2)* partially rescued the dpp phenotype (Fig. 1 c to e). Quantification of the male and female wing area of flies expressing either HH, SEMA1A, or WNT2 verified the visual partial rescue with expression of any of the 3 in females but only WNT2 in males (Fig. 1f). The rescue by HH is very variable in males and females with some strong increases in wing area in females (*P* = 0.008) and with some males strongly rescued, but not found significant overall; however, expression of SEMA1A rescued wing growth in females (*P* = 0.001) but not males (*P* = 1), indicating sexually dimorphic rescue. About a third of the 106 genes identified in 2022 encoding receptor ligand activity were screened, and 3 rescues of the dpp hypomorphic phenotype were identified, suggesting that screening all 106 genes may identify 4 to 8 additional examples of partial rescue and potentially an example(s) of strong rescue. Repeating the search of FlyBase with the GO term “receptor ligand activity” in 2026 increased the number of genes identified to 118, adding to the potential of identifying additional examples of rescue. The high frequency of rescue observed in this screen suggests that screens for rescues of other phenotypes due to the loss of expression of other signaling ligands have a more than reasonable chance of successfully identifying phenotypic nonspecificity as was found in repeated screens for rescue of 8 of 9 distinct TF phenotypes (Percival-Smith 2017; Percival-Smith et al. 2023).

**Fig. 1.**
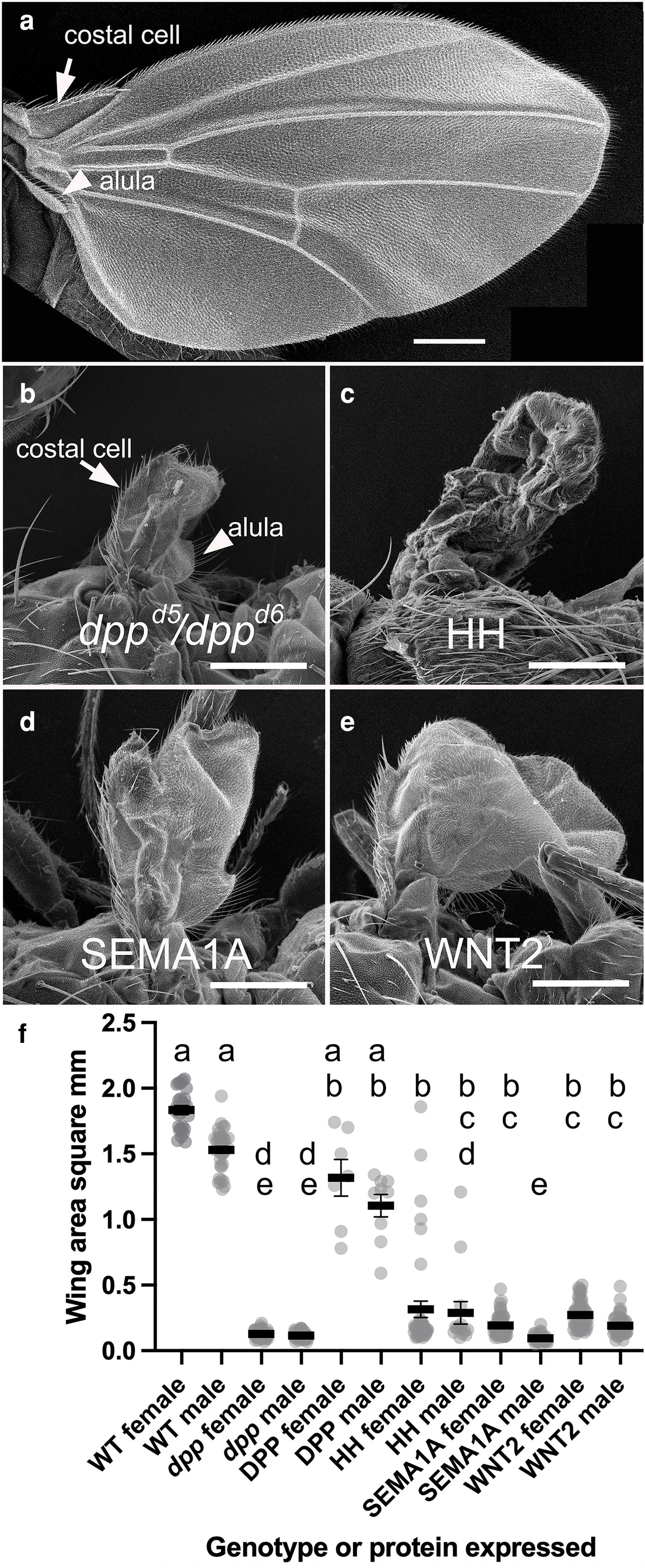
Rescue of the *dpp^d5^/dpp^d6^* hypomorphic wing phenotype by the expression of HH, SEMA-A1, or WNT2. Panels (a to e) are scanning electron micrographs of adult flies that are either (a) wild type, (b) *y w; dpp^d5^/dpp^d6^; dppGAL4*, (c) *y w; dpp^d5^/dpp^d6^; dppGAL4/P{UAShh, w^+^}*, (d) *y w; dpp^d5^/dpp^d6^; dppGAL4/M{UASSema-a1, w^+^}*, or (e) *y w; dpp^d5^/dpp^d6^; dppGAL4/M{UASWnt2, w^+^}*. The proximal distal axis of the wings is left to right in panel (a) and bottom to top in panels (b to e). The scale bar shown in all 5 panels is 200 μm; the magnification in panels (b to e) is the same but reduced for panel (a). The costal cell of the wing is indicated by an arrow and the alula is indicated by an arrowhead in panels (a and b). Panel (f) is a scatter plot of wing area of male and female flies. The mean and SD are indicated. Analysis of the data with an ANOVA of ranks detected differences [H(12) = 325, *P* < 0.0001] and post hoc multiple pair-wise comparisons were performed with Dunn's test. The data that are not different (*P* > 0.05) have the same letter. WT wild type; dpp *y w; dpp^d5^/dpp^d6^; dppGAL4*; DPP *y w; dpp^d5^/dpp^d6^; dppGAL4/P{UASdpp, w+}*; HH *y w; dpp^d5^/dpp^d6^; dppGAL4/P{UAShh, w^+^}*; SEMA-A1 *y w; dpp^d5^/dpp^d6^; dppGAL4/M{UASsema-a1, w^+^}*; and WNT2 *y w; dpp^d5^/dpp^d6^; dppGAL4/M{UASWnt2, w^+^}*.

**Table 1.** Results of the screen for rescue of *dpp^d5^/dpp^d6^*.

		Genotype/phenotype		
		*dpp^d5/d6^;TM* ^a^	*dpp^d5/d6^;UAS* ^b^	
Stock #	Insert	No rescue	No rescue	Rescue
1486	*dpp*	9	0	5
5815	*Ser*	6	3	0
5918	*wg*	5	2	0
8611	*Dl*	8	10	0
26695	*Dl*	20	16	0
26822	*Dl*	0	0	0
38409	*wntD*	14	4	0
51280	*egr*	16	14	0
58426	*Pvf1*	33	8	0
58436	*spi*	18	14	0
63058	*gbb*	13	14	0
63060	*scw*	34	11	0
64108	*Gbp3*	29	5	0
64231	*bnl*	4	6	0
64298	*Wnt5*	28	5	0
65734	*Sema1a*	14	0	5
80708	*Dh44*	0	0	0
80936	*ilp2*	57	19	0
81025	*hh*	22	0	6
91559	*myo*	6	3	0
92603	*trk*	15	4	0
93873	*pyr*	33	26	0
F000939	*Wnt2*	6	0	14
F000997	*Tk*	14	7	0
F001112	*wnt4*	16	8	0
F001322	*Tk*	22	9	0
F001365	*boss*	10	10	0
F002239	*gpb5*	34	15	0
F002274	*CG14069*	0	1	0
F002281	*Dsk*	7	2	0
F002456	*CG31832*	24	13	0
F002663	*wntD*	8	4	0
F002754	*Krn*	18	8	0
F002776	*Eh*	1	1	0
F002826	*Ms*	0	0	0
F002862	*Pvf1*	13	11	0
F003080	*spz5*	10	10	0
F003546	*Wnt6*	2	3	0
F003632	*Dh31*	0	0	0
F003742	*CG3513*	0	0	0
F003794	*AstC*	4	3	0
F003829	*CCHa1*	22	17	0
F003866	*Ilp6*	29	21	0
F003980	*Ilp4*	31	10	0
F004261	*AstA*	5	2	0

^a^ *y w; dpp^d5^/dpp^d6^; dppGAL4/TM6B, Tb*.

^b^ *y w; dpp^d5^/dpp^d6^; dppGAL4/P or M{UASX, w^+^}*.

### Activation of DPP expression as a mechanism for rescue of the hypomorphic dpp phenotype

The role of HH in activating DPP expression required for wing growth and pattern formation is well described (Tabata and Kornberg 1994) and is a potential explanation for the partial rescue of the dpp hypomorphic phenotype. Overexpression of HH with the *dppGAL4* driver may increase expression of *DPP* mRNA from the 2 hemizygous hypomorphic allelic loci *dpp^d5^* and *dpp^d6^*, resulting in increased DPP signaling activity and partial rescue. Therefore, this raises the question of whether WNT2 or SEMA1A also activate DPP expression like HH rather than substituting for DPP. Due to the embryonic lethality of *dpp* null alleles, this question cannot be addressed directly with an analysis of genetic epistasis in adult wing growth. Instead, a gain-of-function phenotype induced by ectopic expression of HH in the wing imaginal disc with the driver *nubbinGAL4* was assessed. Ectopic expression of HH throughout the wing pouch with the *nubbinGAL4* driver resulted in a circular wing with the anterior compartment transformed to posterior identity; the anterior wing margin lost its characteristic rows of bristles and was replaced with a tricomb margin characteristic of the posterior wing margin (Fig. 2c). In addition, vein formation was reduced. Ectopic expression of DPP with *nubbinGAL4* resulted in a small, circular wings with no vein formation, but the anterior compartment was not transformed to posterior identity. The inability of the DPP wing to inflate posteclosion explains its smaller size relative to the HH wing (Fig. 2b). The ectopic expression of HH and DPP induces similar phenotypes, as expected from their sequential function on the same developmental pathway. Ectopic expression of both SEMA1A and WNT2 did not affect wing formation like ectopic expression of either HH or DPP. WNT2 wings were wild type (Fig. 2e and a), while SEMA1A wings had a small vein spur off the posterior cross-vein (Fig. 2d; Jürgens et al. 2024). The ectopic expressions with the *nubbinGAL4* driver do not support an obvious role for WNT2 and SEMA1A, as observed for HH, in activating DPP expression as a mechanism for partial rescue.

**Fig. 2.**
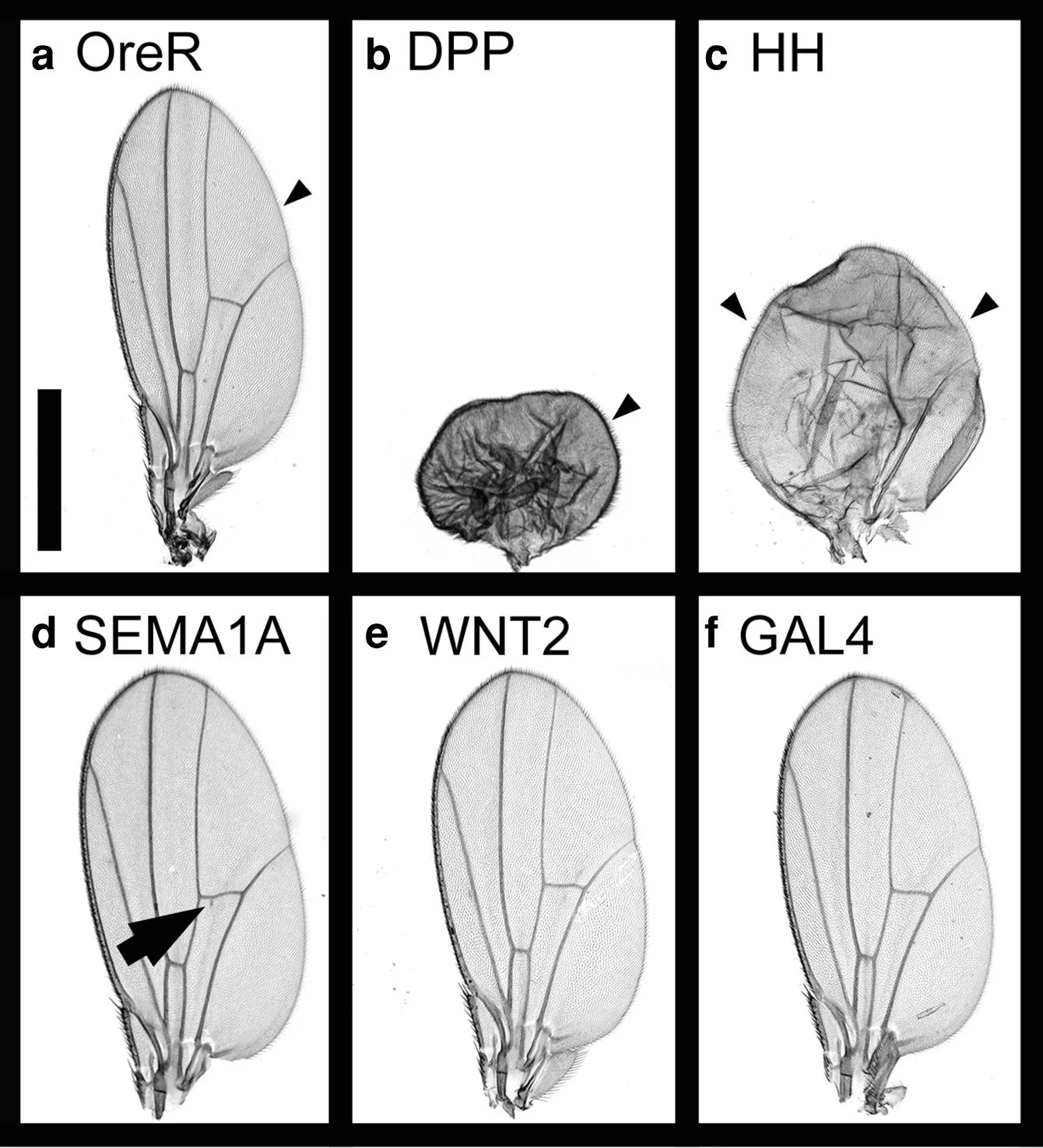
Ectopic expression of DPP, HH, SEMA1A, and WNT2 in the wing disc with the *nubbinGAL4* driver. All panels are female wings. a) OreR wild type flies; b) expressing DPP *y w/w; nubbinGAL4/P{UASdpp, w+}*; c) expressing HH *w/w; nubbinGAL4/P{UAShh, w^+^}*; d) expressing SEMA1A *y w/w; nubbinGAL4/M{UASsema-a1, w^+^}*; e) expressing WNT2 *y w/w; nubbinGAL4/M{UASWnt2, w^+^}*; f) expressing GAL4 from *nubbinGAL4* alone. The bar in panel (a) indicates 500 μm and applies to all panels. The arrowheads in panels (a to c) indicate the row of tricombs characteristic of the posterior wing compartment, and the arrow in panel (d) indicates the vein spur on the posterior cross-vein.

### WNT2/Frizzled4 and SEMA1A/Plexin A requirement in wing formation

The rescue of the hypomorphic dpp phenotype by SEMA1A and WNT2 signaling ligands not resident to the *dpp* locus may occur by the nonresident signaling ligand either having a role along the DPP pathway downstream of DPP or by the ectopic expression of the signaling ligand complementing the loss of DPP expression. If the nonresident signaling ligand has a role in the DPP pathway, the signaling ligand will be required for wing growth, and if the signaling ligand complements the DPP pathway, it will not be required for wing growth.

Both *WNT2* and *SEMA1A* mRNA accumulate in wing imaginal discs (Supplementary Table 3; Sidhu and Percival-Smith 2025). *WNT2* mRNA accumulates in the wing pouch (Fig. 3d; Everetts et al. 2021; Yu et al. 2020). To assess the role of WNT2 in wing development, the wing area of *Wnt2^O^/Wnt2^KO^* null hemizygotic females was measured, and a size decrease was not detected, suggesting that WNT2 does not promote wing growth (Fig. 3j; Kozopas et al. 1998; Yu et al. 2020). Rather, the small increase in wing area suggests that WNT2 is required for suppression/restriction of wing growth. Therefore, WNT2 activity is not downstream/epistatic to DPP in the DPP-dependent wing growth pathway, suggesting that WNT2 substitutes for the loss of DPP. However, it is puzzling how ectopic and likely overexpression of WNT2 with *dppGAL4* results in partial rescue of wing growth when WNT2 is already expressed in the wing pouch and required for restriction of wing growth.

**Fig. 3.**
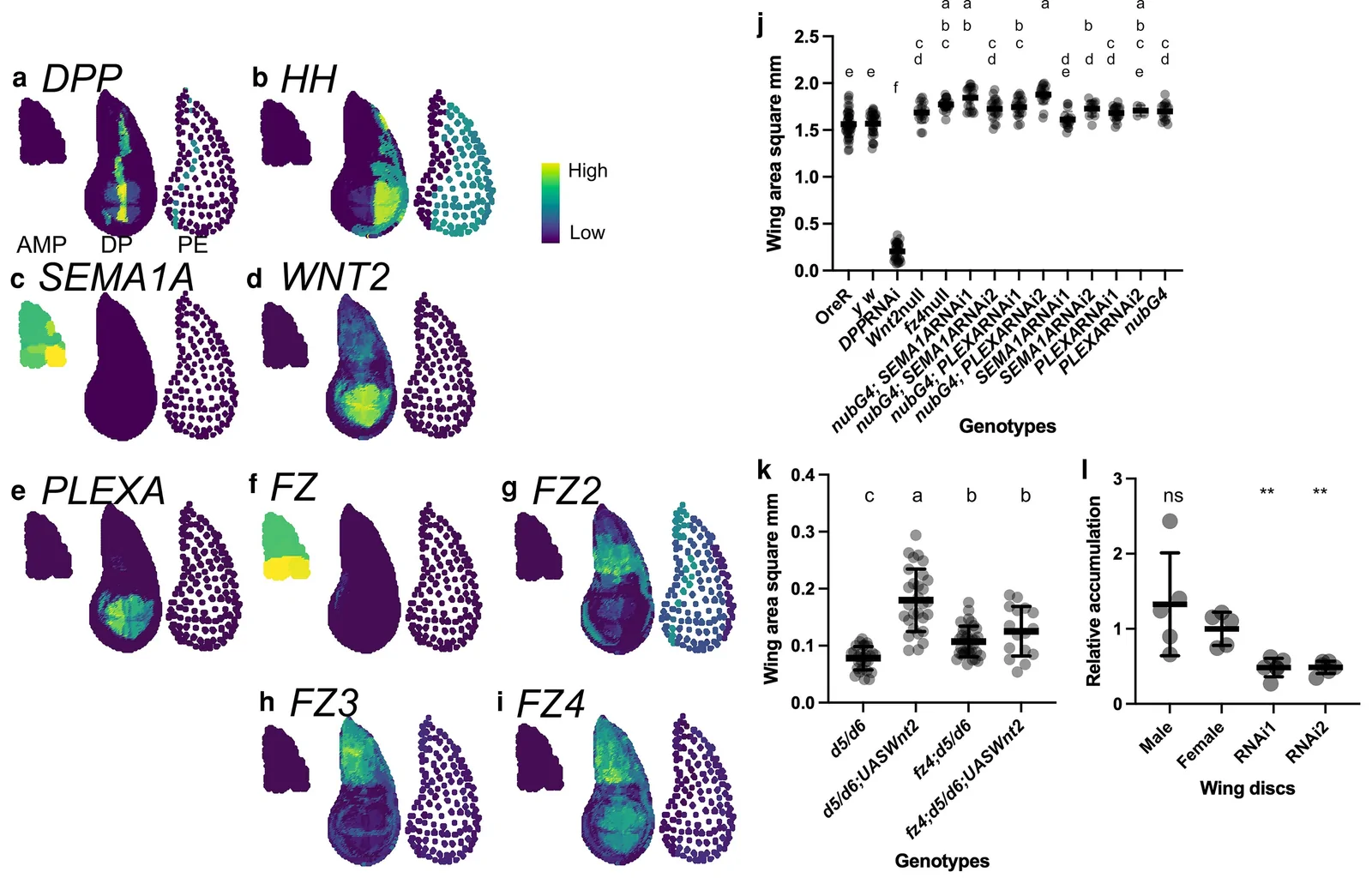
The expression and requirement of SEMA1A and WNT2 for wing growth. Panels (a to i) are virtual in situs generated using the data and method of Everetts et al. (2021). Each panel from left to right shows the cells of the adult AMPs, the disc proper (DP), and the peripodial envelop (PE). a) *DPP* mRNA accumulation, b) *HH* accumulation, c) *SEMA1A* accumulation, d) *WNT2* accumulation, e) *PLEXA* accumulation, f) *FZ* accumulation, g) *FZ2* accumulation, h) *FZ3* accumulation, and i) *FZ4* accumulation. The mRNA accumulation scale is to the right of panel (b). Panel (j) is a scatter plot of the wing areas of female wings assessing the requirements of SEMA1A and WNT2 for wing growth. The wing area is on the *y* axis, and the genotypes are indicated below the *x* axis. Genotypes written in the form *nubG4;XRNAiY* indicate *nubGAL4* driving expression of an *RNAi* molecule; genotypes written *XRNAiY* indicate no expression of GAL4, and *nubG4* indicates expression of GAL4 alone without an *RNA*i molecule. The data was analyzed with an ordinary ANOVA (*F*_13, 276_ = 441; *P* < 0.0001), and the post hoc multiple comparison was performed with Tukey's test. Data with the same letters are not different (*P* > 0.05). *SEMA1A RNAi1 y^1^ v^1^; P{TRiP.JF03231}attP2*; *SEMA1ARNAi2 y^1^ sc v^1^ sev^21^; P{TRiP.HMS01307}attP2*; *PLEXARNAi1 y^1^ sc v^1^ sev^21^; P{TRiP.HM05221}attP2*; and *PLEXARNAi2 y^1^ sc v^1^ sev^21^; P{TRiP.GL01870}attP4* (Supplementary Table 1). Panel (k) is a scatter plot of the wing area of female wings assessing the requirement for FZ4 for WNT2-dependent wing growth. The wing area is on the *y* axis, and the genotypes are indicated below the *x* axis. The data was analyzed with an ordinary ANOVA of log-transformed data [*F*(3, 103) = 37; *P* < 0.0001], and the post hoc multiple comparison was performed with Tukey's test. Data with the same letter are not different (*P* > 0.05). Panel (l) is a scatter plot of relative *PLEXA* mRNA accumulation. The accumulations are relative to the average accumulation in wild-type female wing imaginal discs. The data was analyzed with an ordinary ANOVA of log-transformed data [*F*(3, 18) = 13; *P* = 0.0001], and the post hoc multiple comparison w performed with Dunnett's test. The ** indicate *P* < 0.01 relative to the *PLEXA* accumulation in female wing imaginal discs; ns, not significant (*P* > 0.05). The mean and SD are indicated in panels (j to l).

All the Frizzled (FZ) WNT receptors are expressed in the wing imaginal disc (Supplementary Table 3; Fig. 3f to i) with *FZ4* mRNA accumulating strongly in the wing pouch (Fig. 3i). Inclusion of a *fz4^3–1^* null allele in flies expressing WNT2 in a *dpp^d5^/dpp^d6^* mutant suppressed WNT2-dependent partial wing growth rescue, indicating that FZ4 is an important WNT2 receptor in the wing (Fig. 3k; McElwain et al. 2011). Both FZ4 and WNT2 restrict wing area (Fig. 3j), and *fz4; dpp^d5^/dpp^d6^* wings were larger than *dpp^d5^/dpp^d6^* wings, confirming the requirement of WNT2/FZ4 in suppression of wing growth (Fig. 3k). The requirement for suppression of wing growth by FZ4 in *dpp^d5^/dpp^d6^* wings suggests that WNT2 expression is present in *dpp^d5^/dpp^d6^* developing wing discs. This observation does not provide support for the proposal that DPP expression activates WNT2 expression to serve as a negative feedback factor in suppression of wing growth. Importantly, the requirement of FZ4 indicates that WNT2 does not stimulate DPP-dependent wing growth by weakly binding to the Thickvein Punt co-receptor and weakly activating the DPP pathway.

*SEMA1A* mRNA accumulates in the adult muscle precursor (AMP) cells of wing imaginal discs (Fig. 3c; Everetts et al. 2021). Although *SEMA1A* mRNA accumulates preferentially in AMP cells; nonetheless, its requirement for wing growth was assessed in the wing pouch. Independent RNAi hairpins that target *SEMA1A* mRNA and Dicer2 (DCR2) to increase the effectiveness of RNAi were co-expressed with a *nubbinGAL4* driver in the wing pouch (Dietzl et al. 2007). No requirement of SEM1A for wing growth was found (Fig. 3j). However, this experiment unlikely affected accumulation of *SEMA1A* in the AMP cells, but, interestingly, the SEMA1A receptor, *PLEXA*, mRNA is expressed in the wing pouch (Fig. 3e; Winberg et al. 1998). The accumulation of *PLEXA* mRNA in the wing disc was reduced by 50% with RNAi expression expressed with *nubbinGAL4* from 2 independent RNAis (Fig. 3l). In flies co-expressing DCR2 with the RNAis to increase the effectiveness of RNAi, no reduction in the wing area was observed (Dietzl et al. 2007). The observed increase in wing area in all the knockdown wings is also observed in all the *UASRNAi* stocks and with expression of GAL4 from *nubbinGAL4* alone, indicating that the increase in wing area is likely due to the genetic background and not a requirement for wing growth suppression (Fig. 3j). *PLEXA* mRNA accumulated in both male and female wing imaginal discs ruling out sex-specific accumulation of *PLEXA* mRNA as the explanation for SEMA1A, sex-specific rescue (Fig. 3l). Flies homozygous for loss-of-function *Sema1a* alleles are partially adult viable, develop wings, but are flightless (Kolodkin et al. 1993). These knockdown experiments, accumulation of *SEMA1A* mRNA, and previous analysis of *Sema1a* loss-of-function alleles (Kolodkin et al. 1993) provide no evidence for a role of *SEMA1A/PLEXA* mRNA in wing growth. In summary, expression of WNT2/FZ4 and SEMA1A/Plexin A (PLEXA) is not required for wing growth, and therefore, the mechanism of rescue is likely substitution of DPP by WNT2 and SEMA1A.

### Conclusion

The expression of DPP, HH, SEMA1A, and WNT2 rescues a hypomorphic dpp adult wing phenotype, and the expression of SEMA1A and WNT2 likely substitutes for DPP. These observations are an expectation, but not proof, of the functional redundancy hypothesis of signal transduction terminal TF function. In addition, that phenotypic nonspecificity is observed in 8 of 9 *TF* loci tested so far suggests that phenotypic nonspecificity of signal ligand function will be observed in screens of other *signal ligand* loci (Percival-Smith et al. 2023). The downstream TFs of the wing SEMA1A and WNT2 pathways are presently unknown, but when identified, the TF redundancy hypothesis could be assessed directly. Phenotypic nonspecificity is an evolutionary opportunity, whereas phenotypic specificity is an evolutionary constraint (Percival-Smith 2018). Phenotypic nonspecificity allows the replacement of 1 developmental pathway with another during evolution (Percival-Smith et al. 2023). Phenotypic nonspecificity may be important for how a pathway generally associated with immunity in animals has become associated with dorsoventral patterning in many insect orders (Pechmann et al. 2021). With the observation of phenotypic nonspecificity of both TF and signaling ligand function, there is no reason to assume phenotypic specificity for the requirements of kinases, phosphatases, and RNA-binding proteins. The null hypothesis of the assumption of phenotypic specificity is easily tested in screens for phenotypic nonspecificity.

## Supplementary Material

jkag202_Supplementary_Data

## Data Availability

All fly stocks were obtained from either the Bloomington or FlyORF stock centers (Supplementary Table 1). The authors affirm that all data necessary for confirming the conclusions of this article are represented fully within the article and its tables and figures. Supplemental material available at *G3* online.
