# Supplementary material for: Rescue of a decapentaplegic hypomorphic wing growth phenotype by expression of the signaling ligands Hedgehog, Semaphorin a1, and Wnt homolog 2 in Drosophila melanogaster
Source: G3 (Bethesda). 2026 Jul 27;16(10):jkag202. doi: 10.1093/g3journal/jkag202 (PMC13643765; doi:10.1093/g3journal/jkag202)
Supplement: Supplementary file 1 [file jkag202_supplementary_data.pdf]

Supplemental Tables.

Table of Contents

Table S1 Table of stocks	pages 2-3
Table S2 Table of primers	pages 4
Table S3 Accumulation of normalized counts of mRNAs in wild-type wing imaginal discs extracted from the data of Sidhu and Percival-Smith 2025.	Page 5

Table S1 Table of Stocks

Stock #	Insert/ gene	Origin	Genotype
Stocks for the screen			
1486	*dpp*	BDSC	*y[1] w[*]; P{w[+mC]=UAS-dpp.S}42B.4*
5815	*Ser*	BDSC	*w[*]; P{w[+mC]=UAS-Ser.mg5603}SS1*
5918	*wg*	BDSC	*w[*]; P{w[+mC]=UAS-wg.H.T:HA1}6C*
8611	*Dl*	BDSC	*w[*]; P{w[+mC]=UAS-Delta::GFP}DA55*
26695	*Dl*	BDSC	*w[*]; P{w[+mC]=UAS-Delta.J}3*
26822	*Dl*	BDSC	*w[1118]; TM3, P{w[+mC]=UAS-Delta.Nde.Myc}3, Sb[1]/T(2;3)ap[Xa]*
38409	*wntD*	BDSC	*w[*]; P{w[+mC]=UASp-wntD.M}3*
51280	*egr*	BDSC	*w[1118]; Prn[GAL4-5], P{w+=UAS-eiger.M}Av, P{w[+mC]=tubP-GAL80[ts]}2/TM6B, P{w[+]=tubP-GAL80}OV3, Tb[1]*
58426	*Pvf1*	BDSC	*w[1118]; P{w[+mC]=UASp2-Pvf1.Rb}3*
58436	*spi*	BDSC	*w[*]; P{w[+mC]=UASp-spi.sec}3/TM3, Ser[1]*
63058	*gbb*	BDSC	*y[1] w[*]; P{w[+mC]=UASp-gbb.GFP}33.1*
63060	*scw*	BDSC	*y[1] w[*]; P{w[+mC]=UAS-scw.R}51/TM3, Sb[1] Ser[1]*
64108	*Gbp3*	BDSC	*w[1118]; P{w[+mC]=UAS-Gbp3.HA}1/TM3, Sb[1]*
64231	*bnl*	BDSC	*w[*]; P{w[+mC]=AyGAL4}25, P{w[+mC]=UAS-EGFP}5a.2; P{w[+mC]=UAS-bnl.S}A1-1/TM6B, Tb[1]*
64298	*Wnt5*	BDSC	*w[1118]; P{w[+mC]=UAS-Wnt5.F}201*
65734	*Sema1a*	BDSC	*y[1] w[*]; PBac{y[+mDint2] w[+mC]=UAS-Sema1a.J}VK00033*
80708	*Dh44*	BDSC	*w[*]; P{w[+mC]=UAS-Dh44.Ant}3*
80936	*ilp2*	BDSC	*y[1] w[*]; wg[Sp-1]/CyO; P{w[+mC]=UAS-Ilp2.B}3*
81025	*hh*	BDSC	*w[*]; P{w[+mC]=UAS-hh.EGFP.H}3/TM3, Sb[1]*
91559	*myo*	BDSC	*y[1] w[*]; P{w[+mC]=UAS-myo.F}23/TM3, Sb[1]*
92603	*trk*	BDSC	*trk[1]/CyO; P{w[+mC]=UAS-trk.C}8.4.1*
93873	*pyr*	BDSC	*w[1118]; wg[Sp-1]/CyO, P{ry[+t7.2]=HB-lacZ}GS1; P{w[+mC]=UAS-pyr.K}3/TM3, Sb[1] Ser[1]*
F000939	*Wnt2*	FlyORF	*y w; M{UAS-Wnt2.ORF.3xHA.GW}ZH-86Fb*
F000997	*Tk*	FlyORF	*y w; M{UAS-Tk.ORF.3xHA.g}ZH-86Fb*
F001112	*Wnt4*	FlyORF	*y w; M{UAS-Wnt4.ORF.3xHA.GW}ZH-86Fb*
F001322	*Tk*	FlyORF	*y w; M{UAS-Tk.ORF.g}ZH-86Fb*
F001365	*boss*	FlyORF	*y w; M{UAS-boss.ORF.g}ZH-86Fb*
F002239	*gpb5*	FlyORF	*y w; M{UAS-gpb5.ORF.3xHA.GW}ZH-86Fb*
F002274	*CG14069*	FlyORF	*y w; M{UAS-CG14069.ORF.3xHA.GW}ZH-86Fb*
F002281	*Dsk*	FlyORF	*y w; M{UAS-Dsk.ORF.3xHA.GW}ZH-86Fb*
F002456	*CG31832*	FlyORF	*y w; M{UAS-CG31832.ORF.3xHA.GW}ZH-86Fb*
F002663	*wntD*	FlyORF	*y w; M{UAS-wntD.ORF.3xHA.GW}ZH-86Fb*
F002754	*Krn*	FlyORF	*y w; M{UAS-Krn.ORF.3xHA.GW}ZH-86Fb*

F002776	*Eh*	FlyORF	*y w; M{UAS-Eh.ORF.3xHA.GW}ZH-86Fb*
F002826	*Ms*	FlyORF	*y w; M{UAS-Ms.ORF.3xHA.GW}ZH-86Fb*
F002862	*Pvf1*	FlyORF	*y w; M{UAS-Pvf1.ORF.3xHA.GW}ZH-86Fb*
F003080	*spz5*	FlyORF	*y w; M{UAS-spz5.ORF.3xHA.GW}ZH-86Fb*
F003546	*Wnt6*	FlyORF	*y w; M{UAS-Wnt6.ORF.3xHA.GW}ZH-86Fb*
F003632	*Dh31*	FlyORF	*y w; M{UAS-Dh31.ORF.3xHA.GW}ZH-86Fb*
F003742	*CG3513*	FlyORF	*y w; M{UAS-CG3513.ORF.3xHA.GW}ZH-86Fb*
F003794	*AstC*	FlyORF	*y w; M{UAS-AstC.ORF.3xHA.GW}ZH-86Fb*
F003829	*CCHa1*	FlyORF	*y w; M{UAS-CCHa1.ORF.3xHA.GW}ZH-86Fb*
F003866	*Ilp6*	FlyORF	*y w; M{UAS-Ilp6.ORF.3xHA.GW}ZH-86Fb*
F003980	*Ilp4*	FlyORF	*y w; M{UAS-Ilp.ORF.3xHA.GW}ZH-86Fb*
F004261	*AstA*	FlyORF	*y w; M{UAS-AstA.ORF.3xHA.GW}ZH-86Fb*
Stocks for analysis			
2062	*dpp*	BDSC	*dpp[d6]/CyO*
2071	*dpp*	BDSC	*dpp[d5]/CyO, P{ry[+t7.2]=sevRas1.V12}FK1*
6958	*$Wnt2^{O}$*	BDSC	*Wnt2[O]/CyO, amos[Roi-1]*
24644	*UASDcr2*	BDSC	*P{w[+mC]=UAS-Dcr-2.D}1, w[1118]; betaTub60D[Pin-1]/CyO*
24645	*UASDcr2*	BDSC	*P{w[+mC]=UAS-Dcr-2.D}1, w[1118];Df(3L)Ly, sens[Ly-1]/TM3, Sb[1]*
25782	*dpp*	BDSC	*y[1] v[1]; P{y[+t1.8]=TRiP.JF01371}attP2*
29554	*Sema-a1*	BDSC	*y[1] v[1]; P{y[+t7.7] v[+t1.8]=TRiP.JF03231}attP2*
30483	*PlexA*	BDSC	*y[1] sc[*] v[1] sev[21]; P{y[+t7.7] v[+t1.8]=TRiP.HM05221}attP2*
34320	*Sema-a1*	BDSC	*y[1] sc[*] v[1] sev[21]; P{y[+t7.7] v[+t1.8]=TRiP.HMS01307}attP2*
38412	*$fz4^{3-1}$*	BDSC	*y[1] w[67c23] fz4[3-1]*
38418	*nubGAL4*	BDSC	*w[*]; P{nub-AC-62, P{w[+mC]=UAS-mCherry.NLS}2; P{w[+mC]=UAS-deGradFP}3/TM3, Sb[1] Ser[1]*
67845	*PlexA*	BDSC	*y[1] sc[*] v[1] sev[21]; P{y[+t7.7] v[+t1.8]=TRiP.GL01870}attP40/CyO*
93385	*dppGAL4*	BDSC	*w[*]; P{w[+mW.hs]=GAL4-dpp.blk1}40C.6/TM6B, Tb[1]*
94899	*$Wnt2^{KO}$*	BDSC	*w[*]; TI{3xP3-mCherry=TI}Wnt2[KO]/CyO*

Table S2 Table of primers for qPCR

Gene	Name	Sequence
PlexA	PlexAF	CAGATAGGGCCAAGCATATAC
PlexA	PlexAR	GGGCGGGTCGAAGCCAC
RpL32	RpL32F	CCAGTCGGATCGATATGCTAA
RpL32	RpL32R	GTTCGATCCGTAACCGATGT

Table S3 Accumulation of normalized counts of mRNAs in wild-type wing imaginal discs extracted from data of Sidhu and Percival-Smith 2025.

mRNA	Accumulation counts
HH	883±236
DPP	1070±161
SEMA1A	847±419
PLEXA	7392±1060
WNT2	1127±373
FZ	1999±276
FZ2	9491±1623
FZ3	701±163
FZ4	1079±210
